# Supplementary material for: Causal interplay between lactose intolerance and gut microbiota: a combined bidirectional Mendelian randomization and in vivo validation study
Source: Front Nutr. 2026 Jun 1;13:1803337. doi: 10.3389/fnut.2026.1803337 (PMC13265576; doi:10.3389/fnut.2026.1803337)
Supplement: Supplementary file 1 [file Data_Sheet_1.zip › supplementary materials/Forward/forest plot/ebi-a-GCST90027679.finngen_R12_E4_LACTONAS.pdf]

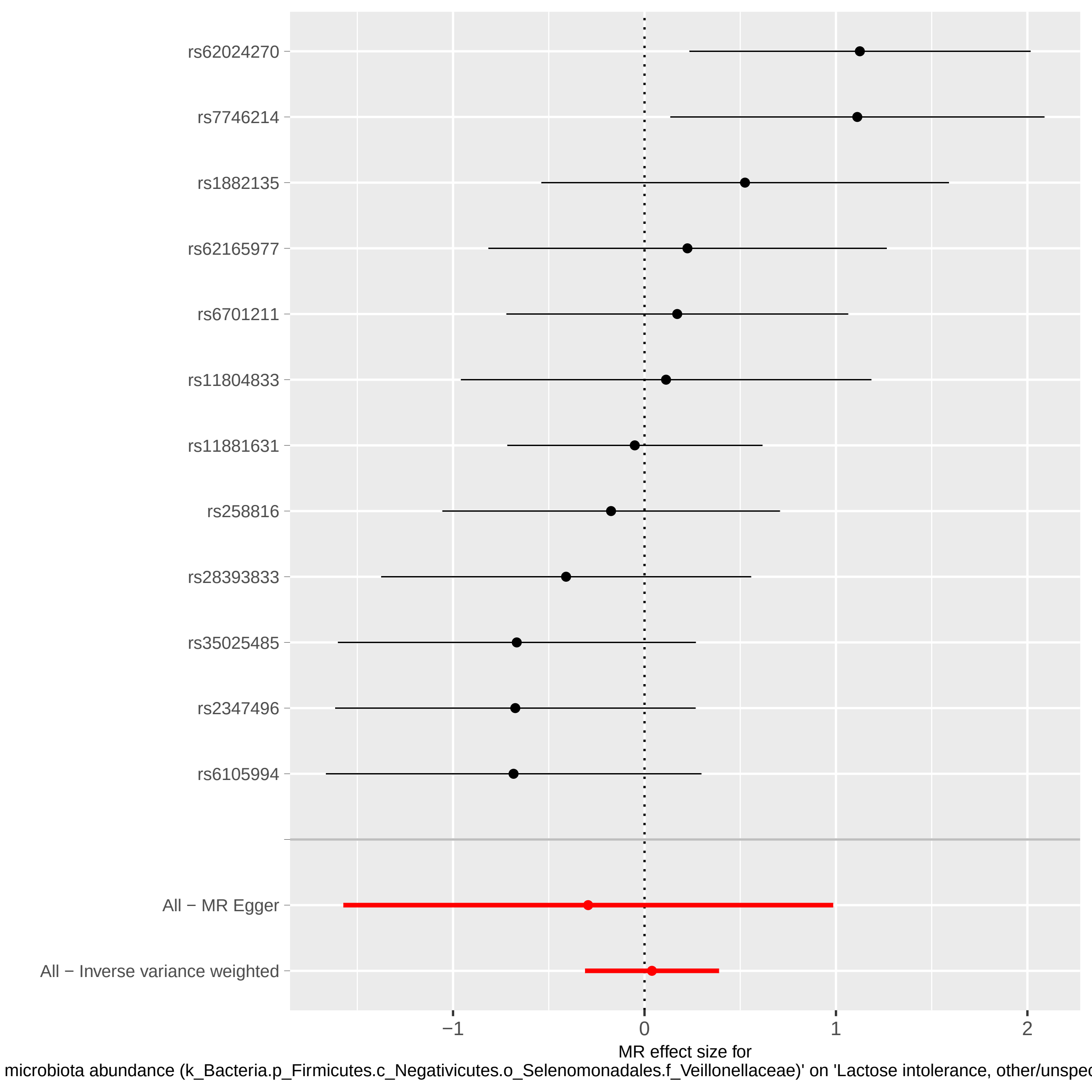
rs62024270
rs7746214
rs1882135
rs62165977
rs6701211
rs11804833
rs11881631
rs258816
rs28393833
rs35025485
rs2347496
rs6105994
All − MR Egger
All − Inverse variance weighted
−1
0
1
2
MR effect size for
microbiota abundance (k_Bacteria.p_Firmicutes.c_Negativicutes.o_Selenomonadales.f_Veillonellaceae)' on 'Lactose intolerance, other/unspec
